# *VviUCC1* Nucleotide Diversity, Linkage Disequilibrium and Association with Rachis Architecture Traits in Grapevine

**DOI:** 10.3390/genes11060598

**Published:** 2020-05-29

**Authors:** Javier Tello, Rafael Torres-Pérez, Timothée Flutre, Jérôme Grimplet, Javier Ibáñez

**Affiliations:** 1 Departamento de Viticultura, Instituto de Ciencias de la Vid y del Vino (CSIC, UR, Gobierno de La Rioja), 26080 Logroño, Spain; rafael.torres@cnb.csic.es (R.T.-P.); jgrimplet@cita-aragon.es (J.G.); javier.ibanez@icvv.es (J.I.); 2 Servicio de Bioinformática para Genómica y Proteómica (BioinfoGP), Centro Nacional de Biotecnología (CNB-CSIC), 28049 Madrid, Spain; 3 Université Paris-Saclay, INRAE, CNRS, AgroParisTech, GQE-Le Moulon, 91190 Gif-sur-Yvette, France; timothee.flutre@inrae.fr; 4 Unidad de Hortofruticultura, Centro de Investigación y Tecnología Agroalimentaria de Aragón (CITA), 50059 Zaragoza, Spain; 5 Instituto Agroalimentario de Aragón-IA2 (CITA-Universidad de Zaragoza), 50059 Zaragoza, Spain

**Keywords:** association genetics, cluster compactness, phytocyanins, rachis lignification, single nucleotide polymorphism (SNP), targeted sequencing, uclacyanins, *Vitis vinifera* L.

## Abstract

Cluster compactness is a trait with high agronomic relevance, affecting crop yield and grape composition. Rachis architecture is a major component of cluster compactness determinism, and is a target trait toward the breeding of grapevine varieties less susceptible to pests and diseases. Although its genetic basis is scarcely understood, a preliminary result indicated a possible involvement of the *VviUCC1* gene. The aim of this study was to characterize the *VviUCC1* gene in grapevine and to test the association between the natural variation observed for a series of rachis architecture traits and the polymorphisms detected in the *VviUCC1* sequence. This gene encodes an uclacyanin plant-specific cell-wall protein involved in fiber formation and/or lignification processes. A high nucleotide diversity in the *VviUCC1* gene promoter and coding regions was observed, but no critical effects were predicted in the protein domains, indicating a high level of conservation of its function in the cultivated grapevine. After correcting statistical models for genetic stratification and linkage disequilibrium effects, marker-trait association results revealed a series of single nucleotide polymorphisms (SNPs) significantly associated with cluster compactness and rachis traits variation. Two of them (Y-984 and K-88) affected two common *cis*-transcriptional regulatory elements, suggesting an effect on phenotype via gene expression regulation. This work reinforces the interest of further studies aiming to reveal the functional effect of the detected *VviUCC1* variants on grapevine rachis architecture.

## 1. Introduction

Current grapevine diversity has been shaped over millennia. Genetic analyses estimate that there are 6000–10,000 different genotypes of cultivated grapevines (*Vitis vinifera* ssp. *sativa*) around the world, originally derived from the wild grape subspecies, *Vitis vinifera* ssp. *sylvestris*, in a process that started *ca*. 8,000 years ago somewhere in the Transcaucasian region [1,2]. During a slow, but constant period of human-driven selection, farmers privileged certain grapevine genotypes that adapted to local climates and fitted their needs to ensure regular fruit production with an appropriate composition for fresh consumption and/or for winemaking, discarding other less-favored genotypes along this process [2]. The different objectives aimed in table and wine grapes led to the artificial selection of different berry and cluster traits, contributing to the wide diversity observable nowadays [3]. Table grape cultivars generally have bigger, heavier, and firmer berries than wine grape cultivars [3,4]. In turn, grapes from wine cultivars used to have higher acidity and sugar content than table grapes, two desirable traits for successful winemaking [3]. Obviously, this divergent selection led to the unconscious selection of some loci, which can now be surveyed through the use of novel high-throughput sequencing and high-resolution phenotyping technologies [5,6,7].

Cluster compactness (or cluster density) was another trait selected during the development of wine and table grape cultivars. Table grape cultivars have, in general, less compact clusters than wine grape cultivars [3,8]. Grapevine varieties with high compact clusters show a major incidence of *Botrytis cinerea* (the causal agent of gray mold on grapes), mainly due to the poorly-aerated and humid microclimate generated in the cluster after rain events [9]. The presence of this ubiquitous fungus might dramatically reduce vine yield and grape quality [10], and has been related to the generation of off-flavors, oxidative damage, and premature aging in wines [11]. Consequently, grape growers are commonly forced to collect grapes from very compact clusters before the fruit has fully ripened [12] and/or to spray plant-protective fungicides at different times to ensure grape production [13], which in turn causes a significant reduction in profit in many viticulture regions [14]. As a result, bunch compactness and related traits are important targets in clonal selection and modern breeding activities to reduce grape production reliance on pesticide treatments aimed to fight major pests and diseases [10,15]. In addition, compact clusters present inner and occluded layers of berries that do not receive the sunlight irradiation needed for an adequate maturation [10]. It hinders the desired homogeneous ripening of all the berries in the cluster at harvest time [16] and impacts wine quality [17].

Cluster compactness is the result of a two-year course in which berry number, berry traits, and rachis traits interact in a complex and dynamic process [8,18]. These three major components are determined at different stages of the grapevine reproductive cycle, so they are affected by many external factors such as temperature, rainfall, and wind [19,20]. Berry number mainly depends on the number of flowers in the inflorescence and the fruit set rate [8], but it can also be affected by abnormal reproductive conditions, like coulure (also known as shatter) or millerandage [21]. Recently, a great diversity has been described in the cultivated grapevine for a series of reproductive performance variables including fruit set rates (which are reported to range from *ca*. 10% to *ca*. 99%) and flower number (from *ca*. 130 to *ca*. 2500 flowers per inflorescence) [21]. This complexity and the low trait heritability might explain the low number of works aimed to reveal the genetic architecture of berry number in grapevine [22,23]. In contrast, berry dimensions have received much more attention, and have been analyzed by quantitative trait loci (QTL) mapping in multiple segregating progenies (see [24] and references therein). At a general level, cell division rate before and after anthesis and cell expansion after anthesis are suggested to be the main factors explaining berry size variation between cultivars [4]. Finally, several studies underline the relevance of rachis traits for cluster compactness determination [8,22,25,26,27]. In general, *Vitis vinifera* L. clusters with long rachis ramifications present a lesser degree of compactness than those with short ramifications [8], and the pre-flowering use of growth stimulators (such as gibberellins) is an effective practice to reduce cluster compactness via cluster rachis elongation [28]. Nevertheless, the genetic determinism of rachis traits in grapevine is poorly understood. By QTL mapping, Marguerit et al. [29] found two QTLs (on linkage groups (LGs) 2 and 10) for peduncle length, and three QTLs (on LGs 2, 3, and 14) for rachis length. Later on, Correa et al. [27] identified 19 QTLs on LGs 5, 8, 9, 14, 17, and 18 for diverse rachis architecture traits (such as peduncle, rachis, and pedicel lengths), whereas Richter et al. [25] found 30 QTLs for cluster architecture traits (including pedicel, rachis, and shoulder lengths), some of them co-localizing in nine QTLs on LGs 1, 2, 3, 10, 12, 17, and 18. Through association mapping, 27 SNPs identified in the sequence of 10 genes located in chromosomes 5, 7, 10, 12 and 18 were found to associate with the length of the first ramification of the rachis in a diversity panel of more than 100 grapevine cultivars [22]. More recently, and through a combination of transcriptome analyses, whole genome sequencing, and transformation experiments, the role of a plant growth-regulating factor (*VvGRF4*) on the elongation of the berry pedicel has been investigated in a series of loose clones of Pinot Noir [26].

Aiming to identify additional candidate genes underpinning grapevine cluster compactness and rachis architecture, a series of comparative transcriptome analyses between Garnacha Tinta and Tempranillo Tinto clones differing in cluster compactness have been performed [30,31]. Among other genes, these analyses revealed a significant differential expression in an *Uclacyanin I*-like gene (*VviUCC1*) between clones with compact and loose clusters [30]. Further analyses identified some *VviUCC1* polymorphisms significantly associated with the variation observed for cluster compactness and the length of the first ramification of the rachis in a diverse collection of wine and table grape cultivars [22]. Uclacyanins, stellacyanins, plantacyanins, and early nodulin-like proteins are four phytocyanins subfamilies. They are ancient blue copper proteins capable of binding to a single copper atom and function as electron transporters [32,33]. Genome-wide identification analyses have revealed a high variation in the number of phytocyanin genes present in different plant species. To cite some examples, 38 phytocyanin genes have been identified in *Arabidopsis thaliana*, 46 in *Solanum lycopersicum*, 74 in *Populus trichocarpa*, and 89 in *Glycine max* [33,34]. In the grapevine genome, 41 phytocyanins have been identified [34]. Their biophysical properties and structural features indicate that phytocyanins are involved in redox reactions occurring during plant primary stress responses and in plant developmental processes including the polymerization of lignin in cell walls and the development of plant fibers [32,35,36]. In fact, multiple reports indicate that phytocyanins have a large influence on plant growth and adaptation to biotic and abiotic stresses [33,34,37]. Several uclacyanins have been shown to be specifically involved in the formation and/or deposition of lignins, as observed in the formation of pea pods [38], during the thickening of radiata pine branches reacting to gravitropism [39], or in the reinforcement of cell walls of Scots pine bark after insect attack [40]. In addition, the presence of a cell wall structural protein domain in uclacyanins suggests the possibility of their association with other structural proteins [41], reinforcing their role in plant development and/or growth processes. Nevertheless, little is known about the role of uclacyanins in grapevine. Genome-wide expression analyses in grapevine cv. Corvina indicates a specific expression of *VviUCC1* in structural organs of the plant, namely roots (from in vitro cultivation), young inflorescences (with single flowers in compact groups), seedlings, and inflorescence rachises [42]. Similarly, the monitoring of the transcriptional development of grapevine inflorescences in cv. Tempranillo Tinto also shows a high expression level of *VviUCC1* in growing young inflorescences [43]. Altogether, these findings suggest a likely role of *VviUCC1* in grapevine inflorescence formation and/or development, which might have an impact on final rachis architecture and therefore on cluster compactness.

In this study, the likely contribution of *VviUCC1* to the phenotypic variation obtained for cluster compactness and a series of rachis architecture traits in a grapevine collection of diverse wine, table, and multi-purpose grape cultivars has been explored. Based on next-generation sequencing data, the *VviUCC1* gene and promoter nucleotide diversity have been characterized, evaluating the degree of linkage disequilibrium (LD) between the polymorphisms detected. In addition, a reduced number of haplotype blocks with little evidence of historic genetic recombination events have been inferred. Finally, a series of association mapping analyses were also performed between the *VviUCC1* gene polymorphisms and the variation observed for cluster compactness and seven rachis architecture traits, considering the phenotypic data of three consecutive seasons and the best linear unbiased prediction (BLUP) values obtained after data modeling.

## 2. Materials and Methods

### 2.1. Plant Material and Phenotyping

A grapevine collection of 114 varieties (including wine, table, and multi-purpose grape cultivars) held at the Grapevine Germplasm Collection of the Instituto de Ciencias de la Vid y del Vino (ICVV, FAO institute code: ESP217) were analyzed (see Table S1 for further details). This dataset includes 111 *Vitis vinifera* L. genotypes and three *Vitis* spp. interspecific crossings grown as hybrid direct producers. Genotypes are maintained as previously detailed [22,44] in two separate plots, “Finca Valdegón” and “Finca La Grajera”, separated by ca. 30 km. These varieties were phenotyped in three consecutive seasons (2011 and 2012 in “Finca Valdegón” and 2013 in “Finca La Grajera”) for a series of traits [8] including cluster compactness (COMP, following the visual O.I.V. descriptor n. 204 [45]) and seven rachis architecture traits: peduncle length (PDULE), rachis length (RALE, measured in 2012 and 2013), pedicel length (PDILE), first rachis ramification length (RM1LE), second rachis ramification length (RM2LE), third rachis ramification length (RM3LE, measured in 2012 and 2013), and fourth rachis ramification length (RM4LE, measured in 2012 and 2013) (Table 1). Rachis traits were measured using standard rules or digital calipers (CD-15DCX, Mitutoyo, Kawasaki, Japan) [8]. In each variety, the mean value of the 10 clusters studied for each trait and year was considered for the association tests. Broad-sense heritability (*H*^2^) was estimated as described elsewhere [22] for the traits described for three seasons (COMP, PDULE, PDILE, RM1LE, and RM2LE), using variance components obtained by the Minimum Norm Quadratic Unbiased Estimator (MINQUE) method by means of SPSS v.22.0 (IBM, Chicago, USA). For these five traits, the BLUP values were estimated through the *lmer* function of the *lme4* package [46] for R v. 3.6.2 to fit a linear mixed-effects model to the experimental data. To this aim, genotypes were used as random variables, and the season (2011, 2012, and 2013) and plot (“Finca Valdegón” and “Finca La Grajera”) values as the covariates. Phenotypic distributions were explored for seasonal data using the *ggplot2* R package, and correlation analyses were performed by means of the *cor* function of the *corrplot* R package using Pearson correlation coefficients (*p* < 0.05).

### 2.2. Genotypic Data and Sequence Analyses

Young and fresh leaves were sampled for each grapevine genotype and frozen at −80 °C. DNA was extracted as previously detailed [22,44]. DNA quality and concentration were assessed by visual comparison with lambda DNA on ethidium bromide-stained agarose gels (0.8%), and by means of a NanoDrop spectrophotometer (Thermo Scientific, Wilmington, USA). Grapevines were genotyped using 25 nuclear SSR loci (*VMC1B11*, *VMC4F31*, *VVIB01*, *VVIH54*, *VVIN16*, *VVIN73*, *VVIP31*, *VVIP60*, *VVIQ52*, *VVIV37*, *VVIV67*, *VVMD21*, *VVMD24*, *VVMD25*, *VVMD32*, *VVMD7*, *ssrVrZAG67*, *VVMD27*, *VVMD5*, *ssrVrZAG29*, *ssrVrZAG62*, *ssrVrZAG112*, *VVS2*, *ssrVrZAG83*, and *VVMD28*) [47,48,49], which were used for structure and kinship matrices estimation, as detailed below. In addition, these markers were used for cultivar identification by means of pairwise comparisons with the grapevine genotypes stored in the ICVV database.

The *VviUCC1* gene (*Vitvi12g00555* in the VCost.v3 gene annotation version [50], corresponding to *VIT_12s0059g02640* in the previous V1 version) was sequenced including 1000 bp in the promoter region according to grapevine 12X V1 gene predictions. Thus, a region of 1971 bp (chr12: 7355541:7357512) was targeted for next-generation sequencing (NGS), performed as previously detailed [44]. Briefly, paired-end libraries with an insert size of ca. 350 bp were sequenced by the Beijing Genomics Institute (BGI, Shenzhen, People’s Republic of China) in an Illumina HiSeq 2000 platform. Resulting reads (90 nucleotides in average) were aligned to the 12X V1 *Vitis vinifera* PN40024 reference genome with Bowtie 2 [51]. Sequence variations (SNPs and insertion/deletions (INDELs)) were detected as previously detailed [22,44], and then filtered to generate a consensus genotype per variety by means of an ad hoc Perl script [44]. Polymorphisms were verified using the Integrative Genomics Viewer (IGV) software [52], and named according to Tello et al. [44]. The likely effect of SNPs and INDELs was predicted with SnpEff v.4.0 [53], and the effect of single amino acid substitution on protein function was predicted by means of SNAP [54] and PROVEAN [55]. The effect of these substitutions on the protein secondary and tertiary structure was explored using the intensive modeling mode of the Phyre2 web portal [56]. The likely effect of the polymorphisms located in the promoter region was evaluated through the in silico detection of putative *cis*-acting regulatory elements, as implemented in PlantCARE [57].

*VviUCC1* haplotypes were inferred using the Partition-Ligation-Expectation-Maximization (PLEM) algorithm implemented in PHASE v.2.1 using default settings [58]. These were tested for recombination using the three alternative algorithms (MaxChi, Chimaera, and 3Seq) available in RDP4 v.4.46 [59]. The software DnaSP v.5.10 [60] was used to estimate the diversity parameters (π and Watterson’s θ) and to run two tests of selective neutrality (Tajima’s D and Fu and Li’s D* tests), considering all the haplotypes detected by PHASE.

Linkage disequilibrium (LD) was calculated considering those polymorphisms with a frequency of the minor allele (MAF) higher than 5%. Thus, standardized disequilibrium coefficients (D’) and squared correlations of allele frequencies (*r*^2^) were calculated for each pair of polymorphisms by means of Haploview v.4.2 [61]. LD blocks (haploblocks) were estimated using the solid spine of the LD algorithm implemented in Haploview with a critical D’ value of 0.80 [62].

Finally, a correlation analysis of expression was performed for *VviUCC1* exploiting the grapevine co-expression database VTC-AGG [63] and using the classification system of the grapevine molecular network VitisNet [64].

### 2.3. Association Analyses

A genotype-phenotype association analysis was performed between polymorphisms with a MAF > 0.05 and seasonal data (2011, 2012, and 2013) and BLUP values available for cluster compactness and rachis architecture traits, using a mixed model (MLM) capable of correcting for population structure (Q) and kinship (K) effects [65]. To this aim, a structure matrix (Q) was calculated by means of STRUCTURE v.2.3.4 [66], as previously detailed [44], but using genetic data on 25 SSRs loci (Zinelabidine et al., in preparation). The same set of markers was used to estimate the pairwise genetic relatedness between genotypes using the method proposed by Wang [67] as implemented in the R package *related* [68] and previously detailed [44]. Association analyses were performed in TASSEL v.3.0 [69] using the P3D (Population Parameters Previously Determined) method with an optimum level of compression.

Aware of the high level of LD between the polymorphisms used in this work, a Bonferroni-corrected factor based on the number of independent tests achieved in this work was applied to set an adjusted p-value threshold to control for false positives. According to Duggal et al. [62], this number of independent tests was determined considering the number of independent markers used, calculated as the number of haplotype blocks (determined with Haploview, as described before) plus all interblock (unlinked) polymorphisms. This approach revealed the presence of three independent tests. Consequently, the association results were evaluated considering three corrected p-values: 1.6 × 10^−2^ (for an α-value of 0.05), 3.3 × 10^−3^ (for α = 0.01), and 3.3 × 10^−4^ (for α = 0.001).

## 3. Results

### 3.1. VviUCC1 Sequence Analysis

**Figure 1:**
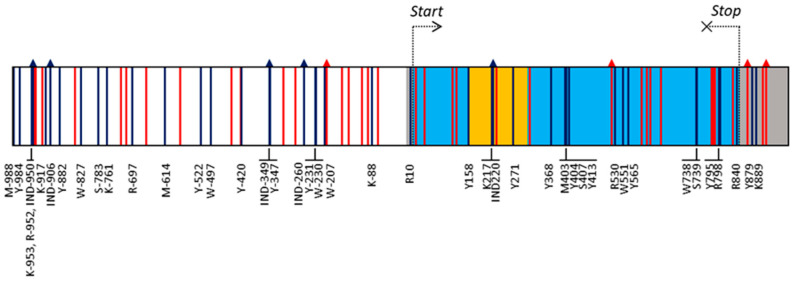
Gene structure and polymorphisms detected in the *VviUCC1* gene sequence. Boxes indicate promoter (white), 5′- and 3′-UTRs (grey), exons (light blue), and intron (yellow). SNPs and INDELs are indicated as vertical lines and vertical arrows, respectively. Their color indicates the frequency of the minor allele (MAF): red < 0.05; dark blue > 0.05. For simplicity, only the name of those polymorphisms with a MAF > 0.05 is shown (the whole list can be found in Table S2).

A region of 1971 bp, corresponding to the *VviUCC1* gene sequence and 1000 bp of the promoter region, was sequenced in a collection of 114 wine, table, and multi-purpose grape varieties. A 100% of coverage of the targeted sequence was achieved in all varieties after alignment with the PN40024 reference genome, with an average coverage depth of 94.1 ± 14.3 reads/nucleotide. BAM files are available at the National Center for Biotechnology Information’s Sequence Read Archive (Accession PRJNA625274). Previous RNAseq analyses [31] confirmed the *VviUCC1* gene structure annotated for the reference PN40024 genome (VCost.v3), consisting of a 5′-UTR (17 bp), two exons (145 and 539 bp) separated by an intron (146 bp), and a 3′-UTR (125 bp) (Figure 1 and Table S2). Thus, the *VviUCC1* gene consists of a coding region of 684 nucleotides that codes for a 228 amino acid protein.

Nucleotide sequence analyses allowed the identification of 80 gene polymorphisms in the set of cultivars analyzed in this work. Of them, nine were INDELs and 71 SNPs (Table S2). Only one INDEL was detected in the coding region of the gene (IND522), which caused a frame shift of 33 nucleotides that generated a protein isoform with 11 additional amino acids. Nevertheless, the frequency of this polymorphism was very low (0.44%), and it was found only in a heterozygous individual for this variant (ESP217-5133, a *Vitis* spp. interspecific hybrid). The other INDELs involved the insertion/deletion of one nucleotide, but for the IND-260, located in the gene promoter and causing the insertion/deletion of 14 nucleotides. The analysis of the SNPs identified in this work revealed the presence of 36 transitions and 35 transversions. Thus, a balanced transition/transversion ratio of 1.03 was found. Among the transitions, A/G and C/T mutations showed an occurrence of 11 and 25 cases, respectively. Regarding the transversions, 8, 11, 4, and 12 cases for A/C, A/T, C/G, and G/T mutations were detected, respectively. Among all the polymorphisms detected, 37 (46.25%) were represented by a rare allele (MAF < 0.05) (Table S2). Considering polymorphisms with a MAF > 0.05 (43 polymorphisms: five INDELs and 38 SNPs; Tables S1 and S2), a higher mutation density in non-coding regions (one polymorphism every 41.5 nucleotides) than in the coding regions (one polymorphism every 57.0 nucleotides) was observed. Interestingly, no mutations with a MAF > 5% were found in the first exon of the gene, but 12 were found in the second exon (Figure 1). SnpEff predicted that five of them generated non-synonymous changes in the coded amino acid: M403 (Pro/His), Y565 (Thr/Ile), S739 (Arg/Thr), Y795 (Leu/Phe), and R798 (Gly/Ser), with a moderate impact on protein function according to the SNAP and PROVEAN results. Of them, M403 is the only mutation potentially affecting the phytocyanin domain of the VviUCC1 protein (according to annotation data, this domain extends from aa16 to aa112). Nevertheless, Phyre2 modeling confidently identified the phytocyanin domain (100% of probability) in the two protein isoforms (Pro- or His- forms) generated by M403, suggesting this mutation does not affect protein function.

Interestingly, in silico analyses revealed one SNP (W738) affecting the first nucleotide of an AGA triplet (in PN40024, coding for Arg) located in the second exon of the *VviUCC1* gene. According to prediction, this substitution generates a premature stop codon (TGA) and therefore a truncated (and probably, non-functional) protein product. This alternative allele of W738 (T) showed a high frequency in the studied collection (65.79%, Table S2). In this set of grapevine varieties, 10 individuals were found to be homozygous for the reference allele (A:A), 58 heterozygous (A:T), and 46 had two putative non-functional alleles (T:T). W738 is adjacent to another polymorphism (S739) that affects the second nucleotide of the same triplet. The joint analysis of these two polymorphisms revealed that 100% of the homozygous individuals for the W738-T allele (T:T) are homozygous for the S739-C allele (C:C), the alternative allele to the reference, generating a TCA codon that will be translated into a serine residue (Table 2). The heterozygous genotypes for W738 (A:T; 58 individuals) were found to be homozygous C:C (33 individuals) or heterozygous C:G (25 individuals) for S739. Among them, only the individuals heterozygous for both SNPs could generate the TGA stop codon (Table 2). Nevertheless, the detailed analysis of the NGS reads of these 25 individuals revealed that the A allele in W738 is always linked with the G allele in S739, and the T allele in W739 with the C allele in S739 (results for Cabernet Franc are shown in Figure S1 as an example). Therefore, only two different alleles are present in these individuals, one producing the AGA codon (reference codon) and another the TCA codon (Table 2). These two codons code for arginine and serine, respectively. Thus, the joint analysis of W738 and S739 revealed that the in silico predicted premature stop codon is never present in the collection of cultivars evaluated.

From the 80 polymorphisms detected in the *VviUCC1* sequence, 46 different haplotypes were inferred including 23 found exclusively in one cultivar (Table S3). No evidence of recombination was found for any of the 46 haplotypes. Six haplotypes showed a frequency above 5% in the set of cultivars analyzed: Hap4 (8.8%), Hap6 (5.3%), Hap16 (10.1%), Hap27 (14.5%), Hap38 (11.4%), and Hap45 (5.3%). On the other hand, nucleotide diversity (π) and Watterson’s θ estimators released values of 0.0076 and 0.0090, respectively. Tajima’s D and Fu and Li’s D* estimators revealed no significant global deviance of neutrality (*p* > 0.05), with values of −0.579 and −1.142, respectively.

The *in silico* analysis of the *VViUCC1* promoter sequence revealed the presence of 69 different *cis*-acting regulatory elements belonging to 19 different classes including four MYB and four MYC binding sites, and a series of common enhancers and/or repressor elements such as 14 CAAT boxes, six TATA boxes, and one TTGACC box. Some of the polymorphisms detected in the promoter affected these binding sites, as observed for the SNP Y-984, which affects a CAAT-box and the SNP K-88, which affects a TATA-box located nearby the transcription start site (Figure S2).

Gene co-expression analysis indicated a series of genes correlated with *VviUCC1* expression levels, possibly representing metabolic partners (Table S4). The top 100 co-expressed genes indicated that *VviUCC1* is commonly expressed with genes related to the phenylalanine and phenylpropanoid metabolic pathways (seven genes), genes coding for membrane transporters (including four ATP-binding cassette (ABC) transporters), genes coding for transcription factors (including seven MYB, two NAC, and two AS2 transcription factors), and genes involved in different hormone signaling pathways (auxin (3), ethylene (2), gibberellins (2) and jasmonate (2) pathways).

### 3.2. VviUCC1 Linkage Disequilibrium (LD) Evaluation

**Figure 2:**
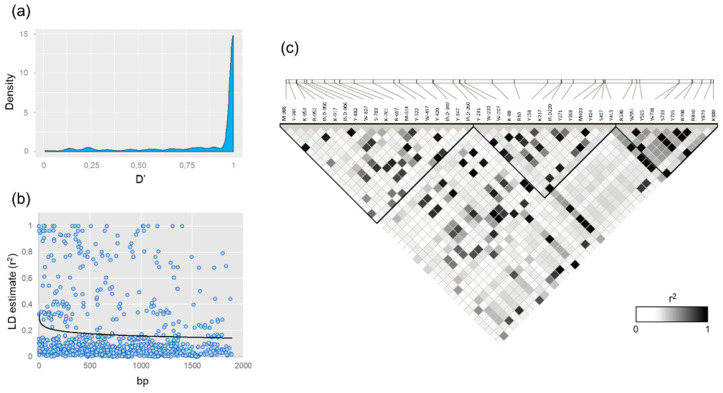
Linkage disequilibrium (LD) evaluation in the *VviUCC1* gene. (**a**) Density plot of the pairwise standardized disequilibrium coefficient (D’) obtained for the 43 *VviUCC1* polymorphisms detected with a MAF > 0.05. (**b**) LD decay plot, using the squared correlations of allele frequencies (*r*^2^) against physical distance (bp). The black line indicates the first-order logarithmic fitting line. (**c**) LD plot representing the magnitude of pairwise *r*^2^ as a white-to-black gradient (see color code). LD blocks are shown as black triangles.

A mean intragenic LD of D’ = 0.87 ± 0.25 was obtained between the 43 polymorphisms found in the *VviUCC1* gene sequence with a MAF above 0.05. As observed in the density plot shown in Figure 2a, many polymorphisms were in high linkage disequilibrium, with 73.7% of the D’ values above 0.90 and 78.6% above 0.80. In addition, many polymorphisms were found to be in perfect LD (D’ = 1; *r*^2^ = 1) even when separated by a large distance (Figure 2b). This is the case of the SNPs pairs Y-420 and K889 (separated by 1309 bp), and Y-522 and Y879 (separated by 1401 bp).

Three haploblocks were predicted from the Haploview-generated LD-plot obtained from the genetic data of 43 *VviUCC1* polymorphisms with a MAF > 0.05 (Figure 2c). No interblock (unlinked) polymorphisms were predicted by the algorithm used for haploblock prediction. The first haploblock included 19 promoter polymorphisms from M-981 to IND-260. It has a mean intrablock LD of D’ = 0.94 ± 0.15 and *r*^2^ = 0.17 ± 0.25. The second haploblock included 14 polymorphisms (from Y-231 to Y413 including four polymorphisms from the promoter, one from the UTR-5′, four from the intron, and five from the second exon) with a mean intrablock LD of D’ = 0.99 ± 0.02 and *r*^2^ = 0.25 ± 0.31. Finally, the third haploblock included 10 SNPs, from R530 to K889 (seven SNPs from the second exon and three from the 3-UTR’). It has a mean intrablock LD of D’ = 0.97 ± 0.11 and *r*^2^ = 0.41 ± 0.32.

### 3.3. VviUCC1 Association Analyses with Cluster Compactness and Rachis Architecture Traits

#### 3.3.1. Phenotypic Diversity for Cluster Compactness and Rachis Architecture Traits

A large phenotypic variation was observed for cluster compactness and seven architecture traits in the grapevine cultivars included in this work (Table 3 and Figure S3). COMP varied from cultivars with very loose/loose clusters (O.I.V. notation = 1/3) to cultivars with very compact clusters (O.I.V. notation = 9). PDULE varied by a 3.5-, 3.7- and 3.2-fold factor in 2011, 2012, and 2013, respectively. RALE varied by a 4.5- and a 5.2-fold factor in 2012 and 2013, respectively. A high variation was also obtained for the lengths of the rachis ramifications measured in this work. For example, RM1LE varied by a 6.4-, 10.7-, and 10.9-fold factor (in 2011, 2012, and 2013, respectively). Finally, PDILE showed the lowest diversity: it varied by a 2.4-, 2.7-, and 2.8-fold factor in 2011, 2012, and 2013, respectively (Table 3). Broad-sense heritability was, in general, higher for rachis architecture traits (*H*^2^ values ranging from 38.9 to 47.1 for PDULE and RM1LE, respectively) than for COMP (*H*^2^ = 32.1). PDILE obtained the lowest *H*^2^ value within the traits here evaluated (*H*^2^ = 31.6).

A highly significant correlation was obtained between the seasonal data for every trait considered as well as when comparing the seasonal data and BLUP values (Figure S4). In addition, the correlations between traits were stable over the seasons. In general, COMP correlated significantly (*p* < 0.05) with all the rachis traits (PDULE, PDILE, RALE, RM1LE, RM2LE, RM3LE, and RM4LE), obtaining negative coefficients in all pairwise correlations. In contrast, rachis traits were positively correlated (*p* < 0.05). A high positive correlation between RM1LE, RM2LE, RM3LE, RM4LE, and RALE was also obtained (Figure S4).

#### 3.3.2. Marker-Trait Association Results

The association analysis carried out between the 43 polymorphisms and eight traits evaluated for three years (using the MLM model with structure and kinship matrices as correction factors) allowed us to identify 35 significant (*p*-value ≤ 1.6 × 10^−2^) associations with seasonal data. Of them, 23 associations were significant at *p*-value ≤ 3.3 × 10^−3^, and six associations at *p*-value ≤ 3.3 × 10^−4^. These associations involved six SNPs: Y-984, Y-231, and K-88 (in perfect LD, D’ = 1; *r*^2^ = 1), Y158, K217, and W551. Although none of them were associated significantly with the same trait for the three seasons evaluated, SNPs Y-984, Y-231/K-88, Y158, and K217 associated during two consecutive seasons (2011 and 2012) with COMP, explaining between 8.9 and 14.1 of trait variance. SNPs Y-984 and Y-231/K-88 were additionally associated with RM1LE in 2011 and 2013, and SNPs Y-984, Y-231/K-88, Y158 and K217 were also associated with RM2LE, RM3LE, and RM4LE in 2013. No significant associations were found for RALE, PDULE, or PDILE. The whole list of significant associations is shown in Table S5, and those markers associated with seasonal data and BLUP values are shown in Table 4.

Considering the association tests performed with BLUP values, 16 significant associations involving ten SNPs were obtained. Y-984 and Y-231/K-88 were found to be associated with three traits (COMP, RM1LE, and RM2LE), Y158 and K217 with COMP, W-497 and R10 (in perfect LD, D’ = 1; *r*^2^= 1), W-207, Y271, and S407 (in perfect LD, D’ = 1; *r*^2^= 1) with PDULE. SNPs associated with COMP, RM1LE, and RM2LE explained between 12.5 and 13.3, 8.3 and 11.2, and 7.4 and 9.9% of BLUP variance, respectively.

## 4. Discussion

Cluster compactness is a complex trait that depends on the interaction of multiple factors [10]. Recent works indicate the relevance of rachis architecture on cluster compactness determination, and features like rachis length, rachis ramifications length, and pedicel length are becoming relevant traits in grapevine breeding and clonal selection activities to reduce cultivar susceptibility to major pests and diseases [15,25]. During the second season of the grapevine reproductive cycle, rachis experiences two main phases that determine its final dimensions. First, rachis elongates rapidly after budbreak and thickens until mid-flowering, almost reaching its final length and 75% of its final cross-sectional area at that time [18,70,71]. After flowering, minor changes in rachis length occurs, and the rachis diameter reaches its final diameter [70]. This second stage is accompanied with an intense process of lignification [72], which provides the mechanical support to the vascular system needed for proper cluster development [73]. In fact, lignin is a major component of grapevine rachises at harvest time, with significant differences on lignin concentration between grapevine cultivars [74]. Despite its relevance, the genetic determinism of rachis architecture in grapevine has been poorly explored, and available knowledge is limited to very few QTLs which, in general, do not colocalize in the mapped populations [22,25,27,29]. Transcriptome profiling is a powerful tool to explore the gene networks and metabolic pathways involved in grapevine development processes, and reports comparing the transcriptome of clones with loose and compact clusters have been used to identify genes likely involved in this phenotypic differentiation [30,31]. Following these works, a differential expression of an *Uclacyanin I*-like gene (*VviUCC1*) between Garnacha Tinta clones with loose and compact clusters was observed during the rapid inflorescence growth occurring prior flowering [30]. Likewise, several works also indicate that this gene is highly expressed during the elongation [18,71] and stiffening [72,73] processes happening during grapevine inflorescences growth before anthesis [42,43]. Altogether, these findings led to the selection of *VviUCC1* as a candidate gene to test its association with the variation observed for cluster compactness and multiple rachis architecture traits in a diverse collection of grapevine cultivars.

The grapevine collection used in this work showed a high diversity for the phenotypic traits evaluated (Table 3), supporting its adequacy for the main objective aimed in this work. As previously reported [8,18,25], a significant negative correlation between cluster compactness and all the rachis traits analyzed in this work was obtained (Figure S4), supporting that shorter rachis elements compress the berries along the rachis and generate more compact clusters. In addition, a high positive correlation between rachis length and the length of all the ramifications of the rachis was found (Figure S4), suggesting a common genetic basis. Interestingly, correlation coefficients between rachis/ramification lengths and peduncle length were slightly lower (Figure S4). In this regard, grapevine peduncle has a different vascular system than the rest of the rachis [73], and its final length is determined earlier than rachis/ramification lengths [18,70]. Accordingly, peduncle and rachis lengths are suggested to be under the control of different loci [25,29]. Similarly, specific QTLs for pedicel length have been reported [25,29], indicating its determinism is (at least, partially) independent to rachis length determinism, and supporting the low correlation values obtained between these traits in the present work (Figure S4).

On the other hand, the *VviUCC1* gene has a high nucleotide diversity, with one polymorphism every 24.6 nucleotides. This level of diversity is higher than the reported for other grapevine genes [75,76], probably because it includes the variation found in the gene promoter, which, as observed in wider frameworks [77], has higher nucleotide diversity than coding regions. In addition, the inclusion of three *Vitis* spp. interspecific hybrid-direct producer genotypes in this work (such as Jacquez or Rubired) might have contributed to increase this diversity value, as previously observed for the grapevine *VvNAC26* gene [44]. In fact, 22 *VviUCC1* polymorphisms were exclusively found in these three hybrid genotypes (Table S1), easily attributed to their non-*vinifera* genetic background. The detailed analysis of the *VviUCC1* gene revealed the presence of 17 non-synonymous mutations causing amino acid changing. However, most of them (11 mutations, 64.7%) were represented by low frequency alleles, suggesting purifying selection processes of deleterious mutations [78]. Thus, considering polymorphisms with a MAF above 0.05, no non-synonymous polymorphisms were detected in the first exon of the gene, and only one (M403) was found to affect the phytocyanin domain (which extends from aa16 to aa112) with a proline/histidine amino acid substitution in the aa86. Nevertheless, the modeling results did not show any effect of this substitution on the phytocyanin domain, suggesting a high level of conservation of the VviUCCl protein function in the cultivated grapevine.

Although *in silico* analyses are useful to predict whether a genetic variant has an impact on the biological function of the coded protein, especially in the big data era, results should be checked considering its biological context to confirm those predicted impacts. Here, *in silico* analyses indicated the presence of a polymorphism (W738) in the open reading frame of *VviUCCl* generating a premature TGA stop codon. Nevertheless, the joint analysis of the allelic diversity observed for this SNP with that obtained for a contiguous non-synonymous SNP (S739) revealed that the predicted TGA stop codon is never present in the grapevine collection (Table 2). Instead, the two non-synonymous SNPs identified in this codon code for three different amino acids, depending on their combination (Arg, Ser, or Thr). A similar finding was previously reported in the gene sequence of an aldehyde dehydrogenase of beech trees [79], which was attributed to different lineages of the detected alleles. The positive association between haplotype frequency and its age [44,75] suggests the allele with the TCA codon (Ser) as the original one (Table 2). In fact, some of the oldest cultivars here analyzed (such as Pinot Noir or Traminer) are homozygous for this allele, supporting this allele as the primal one. Frequency data indicate that this original allele would have experienced a first transversion at W738 to generate the allele with the ACA codon (Thr), which later on would have suffered a second transversion at S739, generating the allele with the AGA codon (Arg).

A common practice in association genetic studies is to set a Bonferroni-corrected threshold to reduce the presence of type-I errors by dividing the original α-value by the number of independent analyses tested, which equals the number of genetic variants if they are unlinked [62]. However, the high intragenic linkage disequilibrium observed in the *VviUCC1* gene invalidates this approach, as previously discussed [22]. Following the approach suggested by Duggal et al. [62], a series of adjusted *p*-value thresholds were set considering that the detected polymorphisms fell within three highly linked haplotype blocks, and consequently they should not be considered as independent genetic variants. In a previous work [22], the significant association of two *VviUCC1* SNPs (Y-231 and K-88) associated with cluster compactness and the length of the first rachis ramification was reported. Here, three additional *VviUCC1* polymorphisms (Y-984, Y158, and K217) were found to be significantly associated with the seasonal variation observed for the length of the ramifications of the rachis and cluster compactness (Table 4). In addition, these five SNPs associated significantly with the BLUP values calculated for these traits, supporting the connection between *VviUCC1* and grapevine rachis architecture traits and cluster compactness. Although SNPs Y158 and K217 are two intronic mutations with presumably little effect on *VviUCC1* function (Figure 1), two associated SNPs (Y-984 and K-88) were found to affect two common *cis*-transcriptional regulatory elements (a CAAT-box and a TATA-box, respectively) (Figure S2). It suggests a possible role of Y-984 and K-88 in the modulation of *VviUCC1* expression, as recently reported for a series of INDELs located in the anthraniloyl-CoA:methanol acyltransferase gene promoter affecting methyl anthranilate accumulation and ‘foxy’ aroma in Concord grapes [80]. Of special interest is K-88, located *ca*. 100 bp upstream of the transcription start site, as proximal sequences of the promoter are suggested to play a core role in gene expression regulation [81].

Uclacyanins are plant-specific cell-wall proteins [32] involved in fiber formation and/or tissue lignification processes [36,38], an activity that is intensified as a response to diverse biotic or abiotic stresses [33,37,39,40]. Cell wall lignification confers stiffness, stability, and strength to vascular plants, enabling the transport of water and solutes through the vascular system and affording physical barriers against environmental factors and phytopathogens [82]. Lignification includes the synthesis of lignin monomers in the phenylpropanoid pathway taking place in the cell cytosol, the transport of these monomers to the cell wall, and their oxidative dehydrogenation and polymerization to form heterogeneous macromolecules that strengthen the plant vascular body [82]. In this complex process, uclacyanins are known to be involved in the oxidative reactions of lignin monomers into lignin polymers in diverse species [32,38]. Accordingly, *VviUCC1* co-expresses with several genes that participate in the lignification process (Table S4). Thus, *VviUCC1* co-expresses with a series of genes of the phenylalanine and phenylpropanoid metabolic pathways including a hydroxycinnamoyl-CoA quinate hydroxycinnamoyl transferase involved in the biosynthesis of lignin monomers, which catalyzes the synthesis of caffeoyl-CoA from *p*-coumaroyl-CoA [83]. *VviUCC1* also co-expresses with multiple genes coding for membrane transporters including a high number of ABC transporters capable of transporting lignin monomers across the cell membrane for their ulterior polymerization [84]. Furthermore, many transcription factors co-express with *VviUCC1*, suggesting a complex regulation process. They include seven MYB transcription factors that could be modulating *VviUCC1* expression through the MYB binding sites found in the *VviUCC1* promoter. In fact, a MYB transcription factor (VvMYB5b) has been shown to contribute to the regulation of the phenylpropanoid pathway in grapevine, with an impact on lignin biosynthesis [85]. Interestingly, *VviUCC1* also co-expresses with multiple genes of the auxin, ethylene, jasmonate, and gibberellin pathways. These hormones play a complex crosstalk to mediate plant defense response against pathogens and abiotic stresses [86], which might promote the expression of transcriptional regulators of the lignin biosynthesis pathway [87].

Taken together, these results suggest that VviUCC1 could be involved in the lignification of the inflorescence rachis that takes place during the elongation and thickening processes occurring along its growth, providing the fundamental structural support needed to bear the weight of the fully developed grapes at the end of the grapevine reproductive cycle, which in some cultivars can be extremely high (above 1 kg in some grape cultivars [5,21]). In addition, lignification processes stiffen the rachis vascular system, allowing the proper transport of water and solutes from the plant to the developing flowers and berries. The vitality of the grapevine rachis is crucial to promote a proper development of berries [73], and structural changes in the vascular system of the rachis can lead to a loss of berry quality and yield [88]. Along this line, it has been reported that the exogenous application of gibberellins at full bloom causes both an elongation and a hardening of the rachis [89,90], the latter caused by an increase in the number of secondary xylem cells and their lignification [90], which promotes an intense berry drop [91] and ultimately might modify cluster compactness.

## 5. Conclusions

The present study provides an in-depth analysis of the nucleotide diversity of the *VviUCC1* gene sequence in the cultivated grapevine. Association analyses indicate that *VviUCC1* may participate in the development of grapevine inflorescence and impact cluster compactness, a trait with major agronomic relevance. The location of the SNPs associated with rachis traits suggests that they might modulate *VviUCC1* expression levels, which could correlate with phenotypic diversity. These findings highlight the need for gene expression studies aimed to assess the functional effect of these SNPs on the determination of grapevine rachis architecture. In addition, this work points out the interest of analyzing the association between the detected *VviUCC1* variants and additional related traits including rachis diameter, rachis stiffness/flexibility, and rachis composition (rachis lignification).

## Figures and Tables

**Table 1:** List of traits evaluated in this work.

Trait	Acronym	Description
Cluster compactness	COMP	Visual compactness of the cluster (O.I.V. rating)
Peduncle length	PDULE	Distance from insertion point on the shoot to the first ramification of the cluster
Rachis length	RALE	Length of the stalk, peduncle excluded
First ramification length	RM1LE	Length of the first ramification of the rachis
Second ramification length	RM2LE	Length of the second ramification of the rachis
Third ramification length	RM3LE	Length of the third ramification of the rachis
Fourth ramification length	RM4LE	Length of the fourth ramification of the rachis
Pedicel length	PDILE	Distance from insertion to ramification (15 independent measurements per cluster)

**Table 2:** Observed genotypic combinations between the *VviUCC1* SNPs W738 and S739 in 114 grapevine cultivars.

W738 Genotype	S739 Genotype	Possible Codon Combinations	Possible Amino Acid Combinations	N Observed Individuals
A:A	C:C	ACA	Thr	4
	G:G	AGA	Arg	2
	C:G	ACA/AGA	Thr/Arg	4
T:T	C:C	TCA	Ser	46
A:T	C:C	ACA/TCA	Thr/Ser	33
	C:G	ACA^1^/TCA/AGA/TGA ^1^	Thr ^2^/Ser/Arg/STOP ^2^	25

^1^ ACA and TGA codons were not observed in the grapevine collection studied. ^2^ The absence of ACA and TGA codons makes impossible the presence of threonine and the formation of a stop codon in these cultivars.

**Table 3:** Mean, standard deviation, maximum (Mx) and minimum (Mn) phenotypic values and broad-sense heritability (*H*^2^) for cluster compactness (COMP) and seven rachis architecture traits (PDULE, RALE, RM1LE, RM2LE, RM3LE, RM4LE, and PDILE).

Trait	2011			2012			2013			*H*^2^ (%) ^1^
	Mean ± s.d.	Mx	Mn	Mean ± s.d.	Mx	Mn	Mean ± s.d.	Mx	Mn	
COMP (OIV rating)	5.6 ± 1.4	9	2.6	4.9 ± 1.5	8.6	1.6	5.4 ± 1.5	8.8	2.4	32.1
PDULE (cm)	4.6 ± 1.1	8.0	2.3	3.8 ± 0.9	7.0	1.9	4.9 ± 1.3	8.3	2.6	38.9
RALE (cm)	*n.a.*	*n.a.*	*n.a.*	11.3 ± 3.8	24.3	5.3	13.7 ± 4.5	27	5.2	-
RM1LE (cm)	4.8 ± 2	10.3	1.6	4.2 ± 2	10.7	1.0	5.2 ± 2.6	13.1	1.2	47.2
RM2LE (cm)	4.2 ± 1.8	10.7	1.4	3.5 ± 1.7	9.2	1.0	4.5 ± 2.3	10.8	1.1	45.1
RM3LE (cm)	*n.a.*	*n.a.*	*n.a.*	2.7 ± 1.4	8.2	0.4	3.2 ± 1.8	8.6	0.6	-
RM4LE (cm)	*n.a.*	*n.a.*	*n.a.*	2.3 ± 1.3	7.5	0.4	2.7 ± 1.5	7.2	0.4	-
PDILE (mm)	6.9 ± 1.2	10.7	4.4	5.6 ± 0.8	8.1	3.0	6.9 ± 1.3	10.7	3.8	31.6

^1^ Broad-sense heritability was calculated for those traits phenotyped in 2011, 2012, and 2013. *n.a*.: not analyzed.

**Table 4:** Association results obtained between *VviUCC1* polymorphisms and cluster compactness and five rachis architecture traits (seasonal data and BLUP values).

Polymorphism	Trait	2011		2012		2013		BLUP	
		*p*-Value ^1^	Trait (%)	*p*-Value ^1^	Trait (%)	*p*-Value ^1^	Trait (%)	*p*-Value ^1^	Trait (%)
Y-984	COMP	8.3 × 10^−4^ **	14.1	3.4 × 10^−3^ *	11.1	2.9 × 10^−2 NS^	-	6.9 × 10^−4^ **	13.2
	RM1LE	4.1 × 10^−2 NS^	-	9.9 × 10^−2 NS^	-	5.3 × 10^−3^ *	10	7.0 × 10^−3^ *	8.3
	RM2LE	1.7 × 10^−2 NS^	-	2.1 × 10^−1 NS^	-	1.2 × 10^−2^ *	8.2	1.0 × 10^−2^ *	7.4
	RM3LE	*n.a*	*n.a*	3.5 × 10^−1 NS^	-	2.3 × 10^−3^ **	11.6	*n.a*	*n.a*
	RM4LE	*n.a*	*n.a*	2.8 × 10^−1 NS^	-	1.6 × 10^−3^ **	12.9	*n.a*	*n.a*
Y-231/K-88	COMP	3.1 × 10^−3^ **	11.3	2.3 × 10^−3^ **	11.9	4.8 × 10^−2 NS^	-	6.9 × 10^−4^ **	13.3
	RM1LE	1.0 × 10^−2^ *	9.3	4.5 × 10^−2 NS^	-	1.3 × 10^−4^ ***	17.8	1.3 × 10^−3^ **	11.2
	RM2LE	1.3 × 10^−2^ *	8.5	7.6 × 10^−2 NS^	-	7.9 × 10^−5^ ***	19	2.5 × 10^−3^ **	9.9
	RM3LE	*n.a*	*n.a*	3.1 × 10^−1 NS^	-	1.8 × 10^−4^ ***	17	*n.a*	*n.a*
	RM4LE	*n.a*	*n.a*	2.5 × 10^−1 NS^	-	3.6 × 10^−4^ **	16.1	*n.a*	*n.a*
Y158	COMP	2.1 × 10^−3^ **	12.1	3.1 × 10^−3^ **	11.3	4.3 × 10^−2 NS^	-	7.7 × 10^−4^ **	13
	RM1LE	6.1 × 10^−2 NS^	-	8.9 × 10^−2 NS^	-	1.7 × 10^−3^ **	12.3	1.7 × 10^−2 NS^	-
	RM2LE	9.1 × 10^−2 NS^	-	1.4 × 10^−1 NS^	-	1.1 × 10^−3^ **	13.1	2.7 × 10^−2 NS^	-
	RM3LE	*n.a*	*n.a*	6.1 × 10^−1 NS^	-	1.4 × 10^−3^ **	12.6	*n.a*	*n.a*
	RM4LE	*n.a*	*n.a*	3.7 × 10^−1 NS^	-	1.8 × 10^−3^ **	12.7	*n.a*	*n.a*
K217	COMP	1.0 × 10^−2^ *	8.9	4.3 × 10^−3^ *	10.7	4.9 × 10^−2 NS^	-	1.0 × 10^−3^ **	12.5
	RM1LE	8.8 × 10^−2 NS^	-	1.2 × 10^−1 NS^	-	4.1 × 10^−3^ *	10.5	3.2 × 10^−2 NS^	-
	RM2LE	4.0 × 10^−2 NS^	-	1.3 × 10^−1 NS^	-	2.6 × 10^−3^ **	11.4	2.8 × 10^−2 NS^	-
	RM3LE	*n.a*	*n.a*	6.1 × 10^−1 NS^	-	3.1 × 10^−3^ **	11	*n.a*	*n.a*
	RM4LE	*n.a*	*n.a*	2.4 × 10^−1 NS^	-	7.0 × 10^−3^ *	9.7	*n.a*	*n.a*

^1, NS^ p-value > 1.6 × 10^−2^; * *p*-value ≤ 1.6 × 10^−2^; ** *p*-value ≤ 3.3 × 10^−3^; *** *p*-value ≤ 3.3 × 10^−4^. *n.a.*: not analyzed.

## Supplementary Materials

The following Supplementary Materials are available online at https://www.mdpi.com/2073-4425/11/6/598/s1, Table S1: Grapevine accessions included in this work and number of clusters described in 2011, 2012, and 2013 for cluster compactness and rachis architecture traits. Genotypes for the 43 polymorphisms with a MAF > 0.05 are indicated for each genotype. Id codes are provided according to the guidelines of the COST action INTEGRAPE CA17111; Table S2: Gene polymorphisms detected along the *VviUCC1* gene sequence and predicted effect; Table S3: *VviUCC1* haplotypes; Table S4: Top 100 co-expressed genes with *VviUCC1* and associated functional annotation; Table S5: Association results obtained between *VviUCC1* polymorphisms and cluster compactness and rachis architecture traits; Figure S1: Alignment of the NGS reads obtained for the grapevine cultivar Cabernet Franc (heterozygous for SNPs W738 and S739) against the PN40024 reference genome. Note that the T and C alleles (in W738 and S739, respectively) are linked as well as A and G alleles (reference alleles); Figure S2: *VviUCC1* gene structure indicating the position of the CAAT-box (in dark blue) and the TATA-box (in pink) affected by SNPs Y-984 and K-88, respectively (both in red). The figure also indicates the position of the four MYB binding sites found in the *VviUCC1* gene promoter (in green); Figure S3: Distribution of cluster compactness and seven rachis architecture traits measured in three consecutive seasons (2011, 2012, and 2013). Cluster compactness (COMP) is shown as OIV rating values; Pedicel length (PDILE) is shown in mm; Peduncle (PDULE), rachis (RALE), first rachis ramification (RM1LE), second rachis ramification (RM2LE), third rachis ramification (RM3LE), and fourth rachis ramification (RM4LE) lengths are shown in cm; Figure S4: Pairwise Pearson’s correlation plot between seasonal data (2011, 2012, and 2013) and BLUP values for cluster compactness and seven rachis architecture traits. Only significant correlations (*p*-value < 0.05) are shown as colored circles according to color code. Circle size is proportional to the correlation coefficient value.
